# Vascular-related NAC-domain 7 directly regulates a broad range of genes for xylem vessel differentiation

**DOI:** 10.1186/1753-6561-5-S7-O37

**Published:** 2011-09-13

**Authors:** Masatoshi Yamaguchi, Nobutaka Mitsuda, Misato Ohtani, Masaru Ohme-Takagi, Ko Kato, Taku Demura

**Affiliations:** 1 Graduate School of Bioscience, NARA Institute of Science and Technology, Japan; 2 National Institute of Advanced Industrial Science and Technology, Japan; 3 RIKEN Biomass Engineering Program, Japan; 4 Graduate School of Bioscience, NARA Institute of Science Technology, Japan and RIKEN Biomass Engineering Program, Japan

## Background

Xylem functions in conduction of water and minerals throughout the plants, and supports the plant body. One of the features of xylem cells is development of secondary wall structure between plasma membrane and (primary) cell wall. Recently, it is expected that knowledge on xylem development can be utilized for application of improvement of the plant biomass, since most portion of wood, which represents one of important sources of woody biomass, is mainly composed of two types of xylem cells, xylem vessels and fiber cells.

Previously we established the *in vitro* transdifferentiation system, in which Arabidopsis suspension cells could synchronously transdifferentiate into xylem vessel elements. A number of genes whose expression is elevated during the transdifferentiation processes have been isolated by using microarray analysis [1]. We revealed that one of the identified genes, which encoded a NAC domain protein, *VND7* (*Vascular-Related NAC Domain Protein7*), plays a pivotal role in promoting the xylem vessel differentiation [1,2].

Recently, to efficiently obtain xylem vessel elements, we used a glucocorticoid-mediated post-translational induction system [3]. The transgenic Arabidopsis plants exhibited transdifferentiation of most of cells into xylem vessel elements, and the plants died. This induction system worked in poplar trees and in suspension cultures of cells from Arabidopsis and tobacco. These data demonstrate that the induction systems controlling VND7 activity can be used as powerful tools for understanding xylem cell differentiation.

## Objectives of the research

Several studies report that VND7 regulates expression of downstream of some transcription factors, suggesting that existence of transcriptional network regulating xylem vessel differentiation. Here, in order to identify direct target genes of VND7, we performed global transcriptome analysis using Arabidopsis transgenic lines in which VND7 activity could be induced posttranslationally.

## Methods

We generated a transgenic Arabidopsis plant expressing *VND7-VP16-GR*[3] driven by *CaMV35S* promoter. *VND7-VP16-GR* and *VP16-GR* seedlings were soaked with water containing 10 µM cycloheximide (CHX), a protein synthesis inhibitor, for 2 hours. After removal of the solution, the seedlings were re-soaked with water containing 10 µM CHX, with or without 10 µM dexamethazone (DEX), for 4 hours. Microarray analysis was performed using GeneChip ATH1 Arabidopsis genome arrays. The effector, reporter, and reference plasmids [4], were delivered into the rosette leaves of Arabidopsis by particle bombardment. After overnight incubation, luciferase activity was assayed. For electrophoretic mobility shift assay (EMSA), promoter fragments were labeled with biotin. The poly-His-tagged N-terminal region of VND7 (His-VND7^1-161^) protein was purified from *Escherichia coli*.

## Results

As shown previously, overexpression of *VND7* induces expression of many genes related to the differentiation of vascular vessels [4]. Microarray analysis revealed that 300 genes are upregulated by more than two-fold in the transgenic *VND7* plant. To identify direct target genes of VND7 among the upregulated genes, we used a glucocorticoid-mediated posttranslational induction system. We generated a transgenic Arabidopsis plant expressing chimera *VND7-VP16-GR* gene under the control of the *CaMV35S* promoter [3]. We subjected *VND7-VP16-GR* seedlings to pre-treatment with CHX, followed by treatment with or without DEX. Microarray analysis using these samples revealed that, among the 300 genes upregulated in the *VND7-YFP* plants, 63 were also upregulated (fold change > 2; FDR < 0.1 [*P* < 0.026]) in the *VND7-VP16-GR* plants in response to DEX treatment. These genes encode a broad range of proteins such as transcription factors, IRREGULAR XYLEM proteins, and proteolytic enzymes, known to be closely associated with xylem vessel formation [5].

To define the promoter region responsible for the upregulation of gene expression by VND7, we carried out transient reporter assays using the *XCP1* promoter sequence. We constructed reporter plasmids by linking various lengths of *XCP1* promoter sequences to a minimal *CaMV 35S* promoter driving the firefly luciferase gene and the *VND7* gene driven by *CaMV 35S* promoter was used as an effector plasmid. We concluded that the region of *XCP1* promoter between residues -211 and -96 is necessary and sufficient for gene expression induced by VND7 [5].

In order to investigate the direct DNA/protein interaction between the *XCP1* promoter sequence and the VND7 protein, we carried out EMSA. Two distinct regions of the *XCP1* promoter were demonstrated to be responsible for VND7 binding. Furthermore, we also showed that VND7 protein binds to several promoter sequences present in candidate direct target genes [5].

## Conclusions

These findings indicated that VND7 upregulates, directly and/or indirectly, many genes involved in a wide range of processes in xylem vessel differentiation. Interestingly, VND7 directly regulates lots of genes involved in programmed cell death, while most of genes controlling secondary cell wall biosynthesis could be regulated through the MYB transcription factors. Further investigation of consensus DNA binding sequences of target genes will help elucidate the regulation of gene expression in xylem vessel formation. To obtain a view of xylem vessel differentiation, we also need to reveal how the direct target genes function during xylem vessel formation.
